# Gene therapy of Hemophilia: Recommendations from the German, Austrian, and Swiss Society for Thrombosis and Haemostasis Research (GTH)

**DOI:** 10.1055/a-1957-4477

**Published:** 2022-12-14

**Authors:** Wolfgang Miesbach, Johannes Oldenburg, Robert Klamroth, Hermann Eichler, Jürgen Koscielny, Susanne Holzhauer, Katharina Holstein, Johanna A. Kremer Hovinga, Lorenzo Alberio, Martin Olivieri, Ralf Knöfler, Christoph Male, Andreas Tiede

**Affiliations:** 1 Medizinische Klinik 2, Institut für Transfusionsmedizin, Universitätsklinikum Frankfurt, Frankfurt, Deutschland; 2 Institute of Experimental Hematology and Transfusion Medicine, University Hospital Bonn, Medical Faculty, University of Bonn, Bonn, Deutschland; 3 Klinik für Innere Medizin – Angiologie und Hämostaseologie, Zentrum für Gefäßmedizin, Vivantes Klinikum im Friedrichshain, Berlin, Deutschland; 4 Institut für Klinische Hämostaseologie und Transfusionsmedizin, Universität und Universitätsklinikum des Saarlandes, Homburg/Saar, Deutschland; 5 Gerinnungsambulanz mit Hämophiliezentrum, Charité, Berlin, Deutschland; 6 Klinik für Pädiatrie m. S. Onkologie und Hämatologie, Charité, Universitätsmedizin, Berlin, Deutschland; 7 II. Medizinische Klinik und Poliklinik, Universitätsklinikum Hamburg-Eppendorf, Hamburg, Deutschland; 8 Universitätsklinik für Hämatologie und Hämatologische Zentrallabor, Universitätsspital Bern, Universität Bern, Bern, Schweiz; 9 Division of Haematology and Haematology Central Laboratory, University Hospital of Lausanne (CHUV) and University of Lausanne (UNIL), Lausanne, Switzerland; 10 Hämophiliezentrum LMU Klinikum - Bereich Pädiatrie, Dr. von Haunerschen Kinderspital, LMU München, München, Deutschland; 11 Universitätsklinikum Dresden Klinik/Poliklinik für Kinder- und Jugendmedizin Bereich Hämatologie, Dresden, Deutschland; 12 Abteilung für Kinder- und Jugendheilkunde, Medizinische Universität Wien, Österreich; 13 Klinik für Hämatologie, Hämostaseologie, Onkologie und Stammzelltransplantation, Medizinische Hochschule Hannover, Hannover, Deutschland

**Keywords:** practice guideline, haemophilia, gene therapy, recommendations, factor VIII, factor IX

## Abstract

Gene therapy has recently become a realistic treatment perspective for patients with hemophilia. Reviewing the literature and our personal experience from clinical trials, we discuss key aspects of hemophilia A and B gene therapy with vectors derived from adeno-associated virus, including predictable results, risks, adverse events, and patient-reported outcomes. Patient selection, informed consent, administration, and monitoring of gene therapy as well as data collection are explained. We also discuss the need for interdisciplinary cooperation with hepatology and other specialties. We emphasize structural and organizational requirements for treatment centers according to the hub-and-spoke model and recommend the use of electronic diaries to ensure safe and timely collection and exchange of data. Electronic diaries will play a key role as a primary source of data for pharmacovigilance, postmarketing clinical studies, national and international registries, as well as health technology and benefit assessment. Reimbursement aspects and the future of gene therapy in adolescents and children are also considered. In a rapidly evolving scientific environment, these recommendations aim to support treatment providers and payers to prepare for the implementation of gene therapy following marketing authorization.

## Introduction

Hemophilia A (HA) and hemophilia B (HB) are X-linked inherited bleeding disorders in which factor VIII (FVIII) and factor IX (FIX), respectively, are absent or exhibit reduced activity. In the year 2018 in Germany, a country with a population of approximately 83 million, 4,240 patients with HA and 785 patients with HB were registered in the German Haemophilia Registry (DHR, *Deutsches Hämophilieregister* ), of whom 2,583 and 403, respectively, are presented with a severe course of the disease. 1 Globally, the number of people with hemophilia is to be expected 1.1 million, 2 of whom a large proportion of patients yet will be undiagnosed. 3

For the treatment of hemophilia, international and national guidelines are available. 4 5 Based on the residual factor activity, the severity of hemophilia is classified into severe (<1 IU/dL or <1% of normal), moderate (1–5 IU/dL or 1–5%), and mild (5–40 IU/dL or 5–40%). 4 In severe and increasingly moderate hemophilia, prophylaxis is the standard of care to reduce the incidence of spontaneous and traumatic bleedings. Prophylaxis with exogenously supplied coagulation factor is administered intravenously and usually by the patients themselves. According to the half-life ( *t*_1/2_ ) of the FVIII factor concentrate used ( *standard half-life* [SHL] in mean 12 hours, *extended half-life* [EHL] 18 hours), prophylaxis in HA is usually given two to three times per week. Because of their longer half-life, FIX SHL (18 hours) is usually given one to two times per week, and FIX EHL ( *t*_1/2_ 100 hours) is even given only one time per 7 to 14 days. 6 7 The aim of prophylaxis with factor concentrates is to maintain the minimum activity (trough level) above 3 to 5%. 4 5

As an alternative to prophylactic factor substitution in HA, the bispecific monoclonal antibody Emicizumab is available, which mimics the function of activated FVIII and is applied subcutaneously at intervals of 1, 2, or 4 weeks. 6 7 In phase 3 clinical trials, additional drugs are being tested for long-term prophylaxis that *act as nonfactor replacement therapy* to balance the impaired coagulation in HA and HB, including monoclonal antibodies to block the *tissue factor pathway inhibitor* (anti-TFPI; Concizumab, Marstacimab) and a *small-interfering* RNA (Fitusiran) that suppresses the biosynthesis of antithrombin in hepatocytes.

Despite advances in the medicinal therapy of HA and HB, there remains still a high need for the improvement of patient-reported outcomes (PROs). These mainly concern the following three areas:

- Bleeding risk: intracranial bleeding (ICB) still causes 10 to 20% of all deaths in hemophilia. One in six patients with an ICB dies, and about half of patients suffer permanent damage. 8
- Joint health: many patients suffer from the so-called hemophilic arthropathy, which can be caused by even a few hemorrhages into a joint and the resulting synovitis. 9 10 It is associated with pain, reduced mobility, premature osteoarthritis, and reduced quality of life. 11
- Participation in life: due to the burdens of therapy, physical limitations, and psychosocial consequences of the disease, the educational opportunities, free choice of occupation as well as sports and social activities are subject to limitations in patients with hemophilia. 12 13

Gene therapy has the potential to effect substantial improvements in PROs in the above-mentioned areas. Thus, consistently higher levels of the clotting factor could prevent the risk of severe bleedings and the development or progression of joint damage. Furthermore, participation in social life could also be improved due to the long-term character of gene therapy.

## Methods

The present recommendations were prepared by the GTH (Gesellschaft für Thrombose- und Hämostaseforschung) working group *Gene Therapy* and are based on a systematic review of the current scientific literature with a focus on clinical trials of gene therapy for HA and HB. Personal experiences and knowledge of the authors from studies on gene therapy were included as well.

### AAV-Based Gene Therapy

**Fig. 1:**
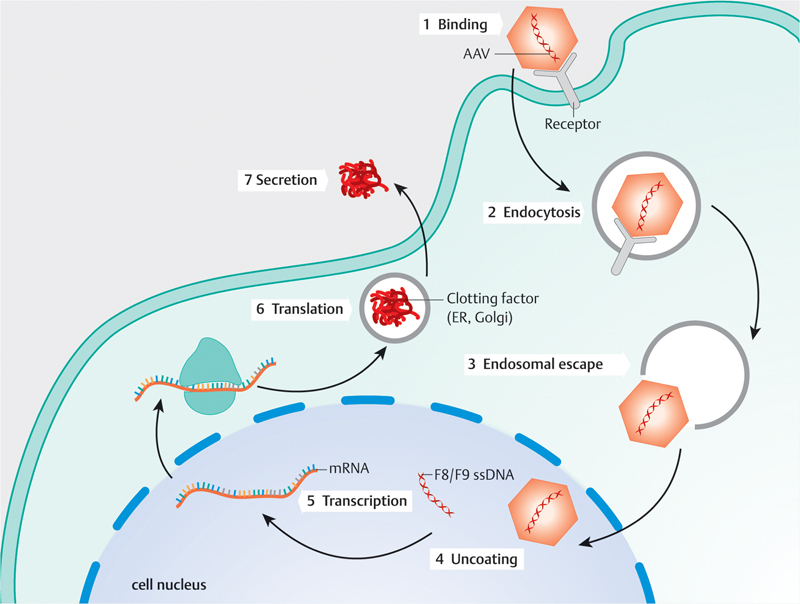
Schematic representation of the mode of action of AAV gene therapy. AAV, adeno-associated virus. (1) The gene therapy is administered intravenously and taken up into the cell by means of endocytosis. (2) After endocytosis, vesicles are formed from the cell membrane (endosomes). (3) After the disintegration of the endosome, the capsid enters the cell nucleus via the nuclear pores. (4) In the cell nucleus, the single-stranded DNA is extracted and released into extrachromosomal episomes. (5) The episomal DNA is transcribed into messenger RNA, which afterward is released into the cytoplasm via the nuclear pores. (6) In the endoplasmic reticulum and Golgi apparatus, the mRNA is translated into proteins. (7) The transgene is secreted into the bloodstream. Abbreviations: AAV = Adeno-associated virus; ssDNA = single-stranded DNA; mRNA = messenger RNA; ER = endoplasmic reticulum.

Currently, the most advanced approach for gene therapy in hemophilia uses recombinant vectors based on the adeno-associated virus (AAV; Fig. 1 ). The nonreplicable vectors contain the complementary DNA (cDNA) for FVIII or FIX, respectively, under the control of a liver-specific promoter. The AAV structural genes and the signal for integration into the genome were removed. The cDNA for the coagulation factor remains predominantly outside the chromosomes, i.e., episomal. Thus, the gene transfer is an additive not a corrective gene therapy. 14

Due to the predominantly episomal destination of the transgene, it was initially thought that it was lost upon division of the hepatocyte. This does not appear to play a major role in liver-healthy adults, as stable factor levels have been observed for up to 8 years in older clinical studies of HB. 15 Preclinical models also showed stable expression for years. Here, it was observed that random vector integration into the genome does occur, but has so far not led to oncogenetically relevant insertional mutagenesis and malignant degeneration. 16 Long-term data from larger patient collectives to answer the question of potential insertional mutagenesis are currently not yet available.

Modifications of the transgene have contributed significantly to the gain in efficiency of gene therapy. Due to the limited packaging capacity, a B-domain-deleted variant of FVIII has been used to date. In vectors for FIX, the R338L variant “Padua” is currently used, which has an approximately 5- to 10-fold increase in specific FIX activity. 17 A difference between gene therapy in HA and HB is also that hepatocytes are the natural site of biosynthesis only for FIX, whereas physiological expression of FVIII actually does not occur in hepatocytes but in liver sinusoidal endothelial cells. This could also be a possible explanation for the different results on long-term expression in gene therapy of HA and HB.

The AAV serotype (variable characteristics of the surface structure) is crucial for the efficiency of hepatocellular transduction and the likelihood for the presence of preformed neutralizing antibodies (NAs) against AAV in the recipient, which may reduce the efficacy of therapy. 18 In Germany, NAs against AAV of different titer levels are expected in 28% (AAV5), 43% (AAV8), and up to 48% (AAV2/6) of patients. 19

To ensure the longest possible therapeutic success and to detect possible side effects, the follow-up after gene therapy is crucial. 20 A few weeks to months after gene transfer, liver enzymes may increase, especially alanine aminotransferase (ALT). This appears to be a cytotoxic immune response against transduced hepatocytes presenting AAV capsid protein on their surface. Nonspecific stimulation of the immune system by CpG oligonucleotides in the cDNA may also play a role. 21 Furthermore, in gene therapy of HA, it is discussed that the nonphysiological production of FVIII in the hepatocyte may lead to ALT elevation. If left untreated, the aforementioned immune responses may lead to loss of transgene expression. A therapy with glucocorticoids, which should be initiated at low ALT elevation, may curb the immune response but is not always effective.

An overview of AAV vectors in clinical development phase 3 is provided in Table 1 .

**Table 1:** AAV-based gene therapy products in phase 3 trials

Manufacturer	Specification	Serotypetransgene	Indication	Phase 3 study	Reference (possibly early study)
Biomarin	Valoctocogene roxaparvovec, BMN270	rAAV5BDD-FVIII-SQ	HA	NCT03370913	Pasi et al 22 Ozelo et al 23
Pfizer/Sangamo	Giroctocogene fitelparvovec, SB − 525	rAAV2/6BDD-FVIII	HA	NCT04370054	Leavitt et al 24
CSL Behring/UniQure	Etranacogene dezaparvovec, AMT − 061	rAAV5FIX-Padua	HB	NCT03569891	Miesbach et al 25
Pfizer/Spark Therapeutics	Fidanacogene elaparvovec, SPK9001	rAAV8 (mod.)FIX-Padua	HB	NCT03861273	George et al 26

Abbreviations: AAV, adeno-associated virus; BDD, B-domain-deleted; FVIII, factor VIII; FIX, factor IX; HA, hemophilia A; HB, hemophilia B; mod., modified; r, recombinant.

### Expected Clinical Benefit and Risks of Gene Therapy

An evidence-based and comprehensible presentation of opportunities and risks is of utmost importance for interested patients and their families, as gene therapy represents a fundamentally new form of therapy. According to the current state of knowledge, AAV gene therapy can be applied only once, since the development of persistent, high-titer NA is to be expected after exposure. Patients therefore make a decision with their consent to gene therapy, whose long-term nature and irrevocability cannot be compared with previous forms of therapy.

#### Expected Benefit

In pivotal studies (single-arm, with intraindividual control), the change in factor activity and bleeding rate are examined as primary endpoints.

In the already fully published phase 3 study with Valoctocogene roxaparvovec, FVIII increased on average to 42% (weeks 49–52). 23 Prophylaxis with factor concentrates was permanently discontinued in almost all participants. Compared with a prospectively considered control period before therapy, the use of factor concentrates decreased by 99%, and the annualized bleeding rate (ABR) decreased by 84%. In case of the 17 participants studied at least 2 years after gene therapy, mean and median FVIII activity levels at weeks 49 to 52 postinfusion were 42.2 ± 50.9% and 23.9%, respectively, as well as 24.4 ± 29.2% and 14.7%, respectively, at week 104, indicating the successive decline in FVIII activity. No inhibitory antibodies to coagulation FVIII occurred.

Final 18-month data from the phase 3 study with Etranacogene dezaparvovec showed FIX increases to 39% (6-month time point) and 37% (18 months) for HB gene therapy. 27 Use of factor concentrates decreased by 97% and the ABR by 64% in this study. Furthermore, patients with pre-existing NA against AAV were not excluded in this study, nevertheless 52 of 54 treated patients achieved a stable FIX expression.

#### Uncertainties and Risks

These benefits are confronted with uncertainties and risks. In the Valoctocogene roxaparvovec phase 3 study, the variance of FVIII expression was high, so that no prediction of achievable factor levels can be made for the individual patient. A few patients even had transiently plasma FVIII levels above normal. There was also a decrease in expression over time, for that reason only indicative data can be given on the durability of therapy. Currently, data on the course of 6 years of the phase 1/2 study (28) and 2 years of the phase 3 study are available. 28

An increase in liver enzymes with the need for immunosuppressive therapy occurred in 85% of patients treated with Valoctocogene roxaparvovec. 23 The duration of treatment with immunosuppressants was in median 230 (22–551) days. Furthermore, 71.8% of patients treated with glucocorticoids developed corticosteroid-typical side effects.

In patients treated with Etranacogene dezaparvovec, 17% had an increase in liver enzymes. 27 The average duration of treatment with corticosteroids was 79 days and completed in all patients by week 26.

Thus, response and duration of the immunosuppressive therapy were individual and varied depending on the study, which is why education about side effects of any longer term corticosteroid medication that may be required must also be provided.

The expression of the transgene is not naturally regulated after gene therapy. Some patients have transiently a significantly increased FVIII or FIX activity. Thromboembolic events have not been reported in the aforementioned phase 3 studies; however, an increased risk cannot be excluded. To date, one thromboembolic event each has been reported in association with increased FVIII activity in the ongoing phase 3 study with Giroctocogene fitelparvovec 29 and with increased FIX activity in a phase 1/2 study with the AAVS3 vector FLT180a. 30

## Selection of Patients

The therapeutic indications are based on the inclusion and exclusion criteria of the phase 3 clinical trials and are the starting point for the selection of potentially eligible patients. They are product-specific and differ, for example, with regard to acceptable NA-AAV titer. Testing for NA against AAV is also product-specific and performed according to the manufacturer's instructions, likely by submission to a specialized laboratory. Liver health, infection status, and history of inhibitors are also critical aspects that must be assessed comprehensively and proactively to ensure optimal patient safety. A successfully treated hepatitis C infection is not a contraindication for conducting a gene therapy, likewise in many cases a well-controlled human immunodeficiency virus (HIV) infection. In case of a hepatitis B infection that has been passed through, the risk of reactivation under possible immunosuppression must be considered.

This also applies especially for potentially hepatotoxic drugs, which should be avoided after gene therapy but may be necessary in the future for patients with certain pre-existing diseases.

In addition to the criteria for admission, other factors play a role in the success of the gene therapy ( Table 2 ). To be suitable for a gene therapy, patients must be willing to accept at least temporary restrictions. They commit themselves to a close-meshed, initially at least weekly monitoring of laboratory values, furthermore to a possibly required intake of immunosuppressants and to extensive documentation. Since vector components can also be excreted in sperm, a safe contraception is obligatory in the first year after gene therapy. Alcohol consumption is discouraged. Thus, gene therapy patients are temporarily exposed to higher stress than patients under usual prophylaxis with factor concentrates or Emicizumab. A high degree of motivation and reliability is required, because once gene therapy has been administered, it cannot be discontinued.

**Table 2:** Aspects of patient selection

Product-specific eligibility criteria	• Age • Severity of hemophilia • Liver health • Immune status in case of HIV infection • Inhibitor status • Neutralizing AAV antibodies
Other aspects	• Motivation of the patient • Assessment of own disease situation • Individual values and preferences • Clinical bleeding propensity • Current or anticipated future difficulties with regular prophylaxis • Current or anticipated future potentially hepatotoxic medication • Compliance to therapy and documentation • Family planning • Foreseeable burdens due to doctor's visits and laboratory monitoring • Possible side effects from immunosuppressants • Cost effectiveness

Abbreviations: AAV, adeno-associated virus; HIV, human immunodeficiency virus.

The necessary compliance can be most likely ensured by optimal informing the patient about the mode of action, expected successes, risks as well as burdens. 31 In addition to official eligibility criteria, the focus is also on personal motivation for gene therapy, which helps to overcome the stress of the first few months. Thereby, the limitations of the previous administered therapy, expectations of gene therapy, and the significance of possible (also future) therapy alternatives should be discussed systematically and with confidence. The aim should be to reach a joint decision between the patient and his or her treating physicians, whereby the discussion should be guided by corresponding suggestions for *shared decision making* . 32 Finally, it should be discussed in which form the usually permanently achieved compensation for disadvantages caused by severe hemophilia in the form of a certain degree of disability can continue to exist after gene therapy.

## Requirements for Hemophilia Centers

To date, one gene therapy for HA has been approved in Europe and one gene therapy for HB in the United States. In addition, two other AAV-based gene therapy approaches are approved in Europe for the treatment of spinal muscular atrophy (SMA) and a form of congenital blindness.

While gene therapy for SMA in infants and young children is administered during an inpatient stay for several days due to the severity of the underlying disease and the age of the patients, 33 gene therapy for hemophilia in trials is performed ambulatory.

The structural and staffing requirements to deliver safe and successful gene therapy are high and will not be able to be provided by all hemophilia centers, even if they already meet the criteria of certified hemophilia centers. 34

On June 14, 2022, the German Federal Joint Committee (Gemeinsamer Bundesausschuss, G-BA) decided to initiate consultation on a quality assurance procedure for gene therapy for hemophilia in the context of the use of “ *advanced therapy medicinal products* ” (ATMP). The ATMP guideline will define quality requirements to the gene therapy in hemophilia centers. Since this is an outpatient therapy, the ATMP guideline must be primarily focused on the outpatient sector. However, a comprehensive care structure that can integrate the entire spectrum of acute medical disciplines when needed will only be possible at large cross-sector sites.

From the requirements it will result that initially only a few centers in Germany, Austria, and Switzerland will be able to perform gene therapy themselves. As “dosing center,” these could also treat patients from referring centers. However, long-term follow-up care will not always be able to take place at the dosing center due to the travel distances involved, which is why the referring center will also take over the function of a “follow-up care center.”

This form of collaboration has been described by the *European Association for Haemophilia and Allied Disorders* and the *European Haemophilia Consortium* as a “hub-and-spoke” model, in which the dosing center is referred to as the “hub” and the referring and follow-up center as the “spoke” ( Table 3 ). 34 This is a flexible and, in principle, modifiable model that adapts to the growing expertise of hemophilia centers. This model is new in hemophilia care in the GTH countries and should be bindingly agreed upon between the participating centers. The hub center should be a hemophilia center that can provide all aspects of hemophilia care. 35

**Table 3:** Suggestion for distribution of responsibilities between Spoke and Hub

Center	Preparation	Therapy execution	Follow-up
Spoke	• Information and education • Indication • Check for eligibility (including neutralizing AAV antibodies) • Request of cost protection • Informed consent for gene therapy • Consent for data collection • Contacting the hub • Transfer to the hub		• Monitoring of liver values and factor activity • Management of late side effects • Data submission to DHR, studies and application-accompanying data collection (AbD)
Hub	• Consultation • Review of eligibility criteria	• Prescription (medical) • Pharmacy contact • Infrastructure for work with genetically modified organisms (GVO) • Ambulatory dosing • Management of infusion reactions and early side effects • Transfer to spoke	• Consultation

Abbreviations: AAV, adeno-associated virus; AbD, application-accompanying data collection; DHR, German Haemophilia Register; GVO, genetically modified organisms.

Prerequisites for performing vector infusion are presented in Table 4 . For the preparation and application of the approved gene therapy agent, no formal authorization according to the Genetic Engineering Act is required, i.e., no S1 or S2 laboratory needs to be established. Nevertheless, it should be noted that in addition to the regulatory and organizational requirements that must be met for vector infusion, professional competence in preparation and follow-up is at least as important for the success of the therapy. Therefore, the requirements for the follow-up center (spoke) are also very high and have to be ensured either by the center itself or by an appropriate cooperation agreement with the hub ( Table 5 ). It is recommended to conclude standardized cooperation agreements in which the respective responsibilities of the cooperating centers and the implementation are clearly defined.

**Table 4:** Aspects of vector infusion

Dosage is based on the actual body weight (vector genomes per kg)
Storage of the pharmaceutically manufactured drug before reconstitution at −60°C
Defrosting and reconstitution under laminar air-flow workbench without UV illumination
Storage of the reconstituted solution for a limited period of time in the refrigerator
Transport to the place of treatment within a cooling box
Administration through commercially available infusion set with microfilter
Discontinuation if intolerant during infusion, administration of antihistamines and glucocorticoids, continuation with a reduced velocity, if possible
Disposal of containers and infusion sets via hospital waste

Abbreviation: UV, ultraviolet.

**Table 5:** Requirements for a hemophilia center for conducting gene therapy

Sector	Requirement	Hub	Spoke
Indication-specific expertise	Certification as HCCC	+	−
	Implementation of a structured education and consent process	+	+ a
Conducting the GT	Request and evaluation of the tests for neutralizing AAV5 antibodies	+	+ a
	Cooperation agreement with pharmacy for ordering, storage, and delivery of the pharmaceutically manufactured drug to the place of treatment	+	−
	Ambulatory performance of the infusion	+	−
	Management of infusion-associated and other early side effects	+	−
Follow-up and documentation	Cooperation with hepatology center	+	+ a
	Daily availability of liver enzyme and appropriate factor activity tests with a turnaround time of less than 24 hours	+	+
	Daily interpretation of laboratory values and transmission of therapy recommendations to the patient	+	+ a
	Management of side effects of immunosuppressants	+	+ a
	Use of an electronic diary for structured implementation, follow-up, and data collection of gene therapy	+	+
	Long-term data collection and transmission to DHR, ÖHR, SHN, and other registries	+	+

Abbreviations: AAV, adeno-associated virus; DHR, German Haemophilia Register; GT, gene therapy; HCCC: Haemophilia Comprehensive Care Centre; ÖHR, Austrian Haemophilia Register; SHN, Swiss Hemophilia Network.

a These tasks may be transferred in whole or in part from a spoke to a hub center within a written cooperation agreement.

## Interdisciplinary Cooperation

The involvement of a hepatology center is necessary to determine the suitability of patients for gene therapy (assessment of infection status and need for therapy of any hepatitis B or C, assessment of fibrosis stage of a chronic liver disease, assessment of potentially required hepatotoxic concomitant medication). Furthermore, in the case of hepatic side effects of gene therapy, the early involvement of hepatology is required. In patients with HIV infection and concomitant antiviral medication, the involvement of immunology or infectiology is required. Close collaboration with other disciplines may also be required, for example, if new concomitant diseases occur after gene therapy or potentially hepatotoxic drugs may be indicated.

## Monitoring and Management of Side Effects after Gene Therapy

Side effects immediately during and after infusion of gene therapy are rare and usually treated ambulatory by the dosing center basis. In exceptional cases, inpatient admission may be necessary, e.g., in the case of protracted or severe symptoms.

The most important side effect is the ALT increase, which usually occurs a few weeks to months after vector infusion. Since this is asymptomatic, regular monitoring of liver enzymes and factor levels should be performed even without clinical suspicion ( Table 6 ). The need for therapy of asymptomatic ALT elevation arises from the observation that it may be associated with loss of factor expression if left untreated. In patients who have never gained factor expression several weeks after vector infusion, therapy of ALT elevation by immunosuppressants is not promising.

**Table 6:** Recommendations for follow-up after gene therapy

Investigation	Frequency
• Alanine aminotransferase (ALT) • FVIII or FIX (with appropriate test, mostly chromogenic)	• <6 months after GT: at least weekly • 6 to 24 months after GT: at least monthly • >2 years after GT: at least six-monthly
• Aspartate aminotransferase (AST) • Glutamate dehydrogenase (GLDH) • Gamma-glutamyl transferase (GGT) • Alkaline phosphatase (AP) • Creatinine kinase (CK) • Cholinesterase • Bilirubin	• On demand to assess differential diagnoses and severity in ALT elevation
• Joint status	• Six-monthly
• Ultrasound and fibroscan of the liver	• At least annually
• Systematic survey on quality of life	• Annually

Abbreviation: GT, gene therapy.

Recommendations on initiation, dosing, and outcome assessment of immunosuppressive therapy in case of an ALT increase are currently still in development and will be investigated in further clinical trials. The GTH working group *Gene Therapy* will monitor the data situation and update its recommendations thereafter. Reference is also made to the manufacturers' prescribing information.

As point of reference, we suggest the start of an immunosuppressive therapy in case of:

- ALT increase above the normal range, or
- ALT increase to more than 1.5 times of the baseline value, or
- Decrease in factor activity by more than one-fifth (20%) of the previous values.

Baseline values of ALT should be determined as the mean of three values before gene therapy, if possible, and prior values of factor activity should be determined as moving average of the last three to five values after gene therapy. Since the determination of laboratory values is subject to strong methodological variations, hence, the analyses should always be preferably performed in the same laboratory using the same equipment and reagents. In case of changes in methodology, baseline or previous values should be re-evaluated. Since transaminases are subject to a circadian rhythm, the determination should be performed at the same time of day, if possible. Deviations from this as well as changes in medication, dietary habits, and alcohol intake must be included in the assessment. Similarly, a late increase in ALT, e.g., more than 1 year after gene therapy, should not necessarily be interpreted as a sign of a cellular immune reaction against transduced cells, but could have other causes.

If an immunosuppressive therapy becomes necessary, the GTH suggests starting with Prednisolone 60 mg per day orally (this dose may be adjusted according to body weight) or the equivalent dose of another glucocorticoid in accordance with phase 3 study protocols. Therapy should initially be given for a period of at least 14 days and then, depending on the course, continued or gradually reduced with further monitoring of laboratory values:

- Start of dose reduction if ALT has decreased by 50% or to the baseline value. Generally, there is no need to wait for complete normalization.
- Reduction to Prednisolone 40, 20, and 10 mg, or to the equivalent dose of another glucocorticoid for 1 week each.
- Then reduction to Prednisolone 7.5, 5, and 2.5 mg or to the equivalent dose of another glucocorticoid for 1 week each, after that discontinue.

If a new ALT increase of more than 1.5-fold occurs during tapering, it is recommended to increase to the last effective dose and retry tapering after 14 days. If Prednisolone therapy of more than 10 mg per day or equivalent is required for more than 1 month, osteoporosis prophylaxis with calcium and vitamin D _3_ should be considered. If risk factors for gastritis or gastrointestinal bleeding exist, a proton pump inhibitor should be given. Prophylaxis of hepatitis B virus (HBV) reactivation in patients with recent HBV infection should be considered.

If tapering of Prednisolone therapy is not successful even in the second attempt, a decision should be made about switching from Prednisolone to Budesonide or the use of a corticosteroid-saving second-line therapy. Tacrolimus, Azathioprine, or Mycophenolate mofetil may be considered for this purpose, given first in combination with the corticosteroid and after that alone. Current studies are investigating the prophylactic use of immunosuppressants to prevent an ALT elevation from the start. 30

An examination for excretion of vector DNA has been performed in clinical studies. Here it is shown that vector components can still be detected in body fluids (urine, stool, saliva, seminal fluid) several months after gene therapy has been performed. 36 In routine practice, testing for this *vector shedding* is not necessary. There is no need for isolation measures in the home, but physical contraception (condoms) is recommended.

To date, tumor diseases have been occurred in four patients after gene therapy. 21 37 38 39 40 The comprehensive clinical, histopathologic, and molecular genetic reappraisal of these cases revealed no evidence of a causal relationship with the gene therapy. Similarly, a comprehensive molecular genetic reappraisal should be initiated in future cases of tumors after gene therapy.

## Use of Electronic Applications

The GTH recommends the use of appropriate electronic systems to meet the special challenges of gene therapy. This applies at first to the eligibility phase, in which essential physical, instrument-based and laboratory examinations must be documented. Checklists can help to collect the required findings and other documentation at the referring center (spoke) before introduction of the patient to the dosing center (hub). 41 These are documented in the application as in a file and, with the patient's consent, shared with the hub center even before a presentation. In this way, unnecessary duplicate examinations and drives to the dispensing center can be reduced.

At the latest with dosing of gene therapy, an electronic documentation system suitable for this purpose should be used, in which all relevant information is documented jointly by the hub center, spoke center, and the patient. Electronic diaries are already common in the documentation of hemophilia treatment and are conscientiously kept by many patients. 39 40 41 42 43 Therefore, it makes sense to actively involve the patient in the documentation of all relevant data. In this way, especially during follow-up, information on bleeding and other PROs can be available to all physicians involved without loss of time and help to provide optimal advice to the patient. The integration of the patient into the documentation system can also help to ensure the information flow to the patient, for example, if the currently recommended concomitant medication is made available in real time and it is visible which center (hub or spoke) is currently in charge of therapy, who is responsible for questions, and who can be reached in case of an emergency. This increases patient safety and promotes long-term compliance following gene therapy. Electronic appointment management and other tools can additionally support this aspect.

An electronic platform shared by the hub, spoke, and patient is particularly useful for managing immunosuppressive therapy. Ideally, both centers should be able to view the relevant laboratory values in real time and assess them for decision making. Visualization of moving averages and percentage deviations from baseline could be useful to support a comprehensible decision-making process.

Not least, electronic information systems for gene therapy management will also be the essential primary source of data needed for registries and trials. These include, but are not limited to:

- Postmarketing surveillance studies by pharmaceutical companies in accordance with the approval requirements of the European Medicines Agency.
- Application-accompanying data collection (AbD) as defined by the G-BA.
- DHR, Austrian Haemophilia Registry (ÖHR), and Swiss Hemophilia Network (SHN) registry.
- Observational studies and international registries such as the World Hemophilia Organization (WFH) registry. 44

In addition to content planning and programming of such an electronic platform, data protection, data security, financing, and contractual regulation between the participating institutions are not insignificant challenges. 45 A lifelong documentation after gene therapy is recommended.

The most advanced is the WFH global Gene Therapy Registry (GTR-WFH).

## Outlook: Financing of Gene Therapy

Gene therapy incurs very high one-time costs at the time of dosing, which are offset by savings in subsequent years. Clinical studies show that the factor consumption already decreases by up to 98% in the first year after gene therapy.

The gene therapy is approved as an outpatient therapy and as such reimbursable. Within the framework of the statutory health insurance (SHI) in Germany, health insurance funds have the option to conclude rebate contracts with the pharmaceutical companies according to SGB V § 130a and thereby implement a “pay-for-performance” (P4P) reimbursement model according to SGB V § 130a (8) (measurable therapeutic outcomes).

On the part of medical care providers, the preparation, implementation, and follow-up of gene therapy are associated with considerably higher costs than the usual hemophilia care. The quality of treatment, which is crucial for the success of therapy, should be agreed upon and remunerated through care contracts, for which § 132i SGB V can provide a framework. Alternatively, care contracts according to § 140a SGB V “Special care” can be considered. Adequate financing models can compensate for the lack of representation of medical care services in the doctors' fee scale within the SHI scheme (Einheitlicher Bewertungsmaßstab, EBM) or in the German medical fee schedule for physicians (Gebührenordnung für Ärzte, GOÄ).

## Outlook: Pediatrics

Although gene therapy trials in childhood have been conducted since the 1990s for rare monogenic and potentially fatal diseases, complications with malignant degeneration and deaths have led to fears and concerns about this form of therapy in affected families, some of which have negative connotations. In recent years, success has been achieved with AAV-based gene therapy for SMA and with lentiviral gene therapy of hematopoietic stem cells for the treatment of transfusion-dependent thalassemia.

In hemophilia, studies for the treatment of children and adolescents with AAV vectors are being planned. Some limitations such as the high prevalence of NA against AAV and the incomplete growth of the liver in early childhood have to be considered. A repeat in AAV gene therapy later in life is not possible according to current knowledge.

In the context of *shared decision making* , not only the patients but also the child's legal custodies require understandable information and advice with regard to the gene therapy. Informing the custodies of underage patients is particularly challenging because proxy decisions have to be made for a minor. Nevertheless, the prospect of an early gene therapy for hemophilia is also associated with the hope of correcting the physical and psychosocial disadvantages of the disease described above in a particularly sustainable way.

## Summary and Outlook

Gene therapy has the potential to outperform patient-relevant endpoints of existing therapies. However, there remains a high variability of response and sometimes a high rate of side effects with ALT elevation and the need for immunosuppressive therapy. Therefore, selection and counselling of appropriate patients are of great importance. Thorough and effective management of side effects, especially elevation of liver enzymes, is critical to the success of therapy. New requirements are formulated for hemophilia centers by quality guidelines, which can be met by cooperation of centers (hub-and-spoke model) and interdisciplinary collaboration. In addition, the cooperation of the centers involved and the involvement of the patient will be supported by modern electronic systems. At the same time, they will help to collect important data longitudinally, which will help to answer open questions and to evaluate the benefits of gene therapy. Gene therapy also poses novel requirements for SHI funding. For pediatric hemophilia patients, gene therapy is not an option today, although clinical trials are being planned.
