# Erratum: Gentherapie der Hämophilie: Empfehlung der Gesellschaft für Thrombose- und Hämostaseforschung (GTH)

**DOI:** 10.1055/s-0042-1760262

**Published:** 2022-12-21

**Authors:** Wolfgang Miesbach, Johannes Oldenburg, Robert Klamroth, Hermann Eichler, Jürgen Koscielny, Susanne Holzhauer, Katharina Holstein, Johanna A. Kremer Hovinga, Lorenzo Alberio, Martin Olivieri, Ralf Knöfler, Christoph Male, Andreas Tiede

**Affiliations:** 1 Medizinische Klinik 2, Institut für Transfusionsmedizin, Universitätsklinikum Frankfurt, Frankfurt, Deutschland; 2 Institute of Experimental Hematology and Transfusion Medicine, University Hospital Bonn, Medical Faculty, University of Bonn, Bonn, Deutschland; 3 Klinik für Innere Medizin – Angiologie und Hämostaseologie, Zentrum für Gefäßmedizin, Vivantes Klinikum im Friedrichshain, Berlin, Deutschland; 4 Institut für Klinische Hämostaseologie und Transfusionsmedizin, Universität und Universitätsklinikum des Saarlandes, Homburg/Saar, Deutschland; 5 Gerinnungsambulanz mit Hämophiliezentrum, Charité, Berlin, Deutschland; 6 Klinik für Pädiatrie m. S. Onkologie und Hämatologie, Charité, Universitätsmedizin, Berlin, Deutschland; 7 II. Medizinische Klinik und Poliklinik, Universitätsklinikum Hamburg-Eppendorf, Hamburg, Deutschland; 8 Universitätsklinik für Hämatologie und Hämatologische Zentrallabor, Universitätsspital Bern, Universität Bern, Bern, Schweiz; 9 Division of Haematology and Haematology Central Laboratory, University Hospital of Lausanne (CHUV) and University of Lausanne (UNIL), Lausanne, Switzerland; 10 Hämophiliezentrum LMU Klinikum - Bereich Pädiatrie, Dr. von Haunerschen Kinderspital, LMU München, München, Deutschland; 11 Universitätsklinikum Dresden Klinik/Poliklinik für Kinder- und Jugendmedizin Bereich Hämatologie, Dresden, Deutschland; 12 Abteilung für Kinder- und Jugendheilkunde, Medizinische Universität Wien, Österreich; 13 Klinik für Hämatologie, Hämostaseologie, Onkologie und Stammzelltransplantation, Medizinische Hochschule Hannover, Hannover, Deutschland

Hamostaseologie 2022. DOI 10.1055/a-1957-4477 .

Published online: 2022-12-14.

This article was subsequently published as an open access article.

