# Supplementary material for: Gene therapy of Hemophilia: Recommendations from the German, Austrian, and Swiss Society for Thrombosis and Haemostasis Research (GTH)
Source: Hamostaseologie. 2022 Dec 14;43(3):196–207. doi: 10.1055/a-1957-4477 (PMC10281774; doi:10.1055/a-1957-4477)
Supplement: Related texts

# Research article: Gentherapie der Hämophilie: Empfehlung der Gesellschaft für Thrombose- und Hämostaseforschung (GTH)

**Keywords:** Praxisleitlinie, Hämophilie, Gentherapie, Empfehlungen, Faktor VIII, Faktor IX

## Abstract

Die Gentherapie hat sich in den vergangenen Jahren zu einer realistischen Behandlungsoption für Patienten mit Hämophilie entwickelt. Unter Berücksichtigung der verfügbaren Literatur und der persönlichen Erfahrungen aus klinischen Studien diskutieren wir hier wesentliche Aspekte der Gentherapie der Hämophilie A und B mit Vektoren auf Basis des Adeno-assoziierten Virus (AAV) einschließlich zu erwartender Ergebnisse, Risiken, Nebenwirkungen und Auswirkungen auf patientenrelevante Endpunkte. Überlegungen zur Auswahl von Patienten, zum Aufklärungs- und Einwilligungsprozess, zur ambulanten Durchführung und Überwachung der Gentherapie sowie zur Erfassung von Daten werden dargestellt. Die Notwendigkeit interdisziplinärer Zusammenarbeit mit der Hepatologie und anderen Fachgruppen wird dargelegt. Besondere Berücksichtigung finden strukturelle und organisatorische Voraussetzungen der Zentren, die Gentherapie durchführen (Dosierungszentren oder „Hub“), und der heimatnahen Zentren, die Patienten der Gentherapie zuführen und die Nachsorge übernehmen („Spoke“). Wir geben eine Empfehlung zur Nutzung einer elektronischen Plattform zum sicheren und verzögerungsfreien Datenaustausch zwischen diesen Zentren und den Patienten. Diese elektronische Plattform wird auch die Primärdatenquelle sein für Pharmakovigilanz, Studien, nationale und internationale Register sowie für die frühe Nutzenbewertung dieser neuen Therapieform. Überlegungen zur Finanzierung der Gentherapie sowie zur möglichen zukünftigen Nutzung bei Jugendlichen und Kindern werden diskutiert. In einem sich rasch entwickelnden wissenschaftlichen Umfeld sollen diese Empfehlungen den betreuenden Einrichtungen und Kostenträgern erste Orientierung in Vorbereitung auf die Anwendung der Gentherapie nach ihrer Zulassung geben.

## Einleitung

Die Hämophilie A (HA) und die Hämophilie B (HB) sind X-chromosomal vererbte Erkrankungen der Blutgerinnung, bei denen Faktor VIII (FVIII) bzw. Faktor IX (FIX) fehlen oder eine verminderte Aktivität ausweisen. Im Jahr 2018 waren in Deutschland, einem Land mit etwa 83 Millionen Einwohnern, 4240 Patienten mit HA und 785 Patienten mit HB im Deutschen Hämophilieregister (DHR) erfasst, von denen 2583 bzw. 403 eine schwere Verlaufsform aufwiesen. 1 Weltweit ist eine Zahl von 1,1 Millionen Menschen mit Hämophilie zu erwarten, 2 von denen ein großer Teil jedoch nicht diagnostiziert ist. 3

Zur Therapie der Hämophilie existieren internationale und nationale Leitlinien. 4 5 Der Schweregrad der Hämophilie wird anhand der residualen Faktoraktivität in schwer (<1 IE/dl bzw. <1% der Norm), mittelschwer (1-5 IE/dl bzw. 1-5%) und mild (5-40 IE/dl bzw. 5-40%) unterteilt. 4 Bei schwerer und zunehmend auch bei mittelschwerer Hämophilie ist eine Prophylaxe der Therapiestandard, um spontane und traumatische Blutungen in ihrer Häufigkeit zu reduzieren. Die Prophylaxe mit exogen zugeführtem Gerinnungsfaktor erfolgt intravenös und in der Regel durch die Patienten selbst. Gemäß der Halbwertszeit (t1/2) des verwendeten FVIII-Faktorkonzentrats ( *standard half-life* [SHL] im Mittel 12 h, *extended half-life* [EHL] 18 h) erfolgt die Prophylaxe bei HA meist 2- bis 3-mal pro Woche. Aufgrund der längeren Halbwertszeit wird FIX SHL (18 h) meist 1- bis 2-mal pro Woche, FIX EHL (t1/2 100h) sogar nur 1-mal pro 7-14 Tage gegeben. 6 Ziel der Prophylaxe mit Faktorkonzentraten ist es, die minimale Aktivität (Talspiegel) oberhalb von 3-5% zu halten. 4 5

Als Alternative zur prophylaktischen Faktorsubstitution bei HA steht der bispezifische monoklonale Antikörper Emicizumab zur Verfügung, der die Funktion von aktiviertem FVIII nachahmt und in Intervallen von 1, 2 oder 4 Wochen subkutan appliziert wird. 7 In klinischen Phase-3-Studien werden weitere Medikamente für die langfristige Prophylaxe geprüft, die als *non-factor replacement therapy* (NFRT) balancierend in die gestörte Gerinnung bei HA und HB eingreifen, darunter monoklonale Antikörper zur Blockade des *tissue factor pathway inhibitor* (anti-TFPI; Concizumab, Marstacimab) und eine *small interfering* RNA (siRNA; Fitusiran), die die Biosynthese von Antithrombin in Hepatozyten unterdrückt.

Trotz der Fortschritte in der medikamentösen Behandlung von HA und HB gibt es weiterhin einen hohen Bedarf zur Verbesserung patientenrelevanter Endpunkte (PRO, *patient reported outcomes* ). Diese betreffen im Wesentlichen drei Bereiche:

1. Blutungsrisiko: Intrakranielle Blutungen (ICB) verursachen noch immer 10-20% aller Todesfälle bei Hämophilie. Jeder sechste Patient mit einer ICB verstirbt, etwa die Hälfte erleiden bleibende Schäden. 8
2. Gelenkgesundheit: Viele Patienten leiden an der sog. hämophilen Arthropathie, die schon durch wenige Einblutungen in ein Gelenk und die daraus resultierende Synovitis ausgelöst werden kann. 9 10 Sie ist mit Schmerzen, reduzierter Mobilität, vorzeitiger Arthrose und eingeschränkter Lebensqualität vergesellschaftet. 11
3. Teilhabe: Aufgrund von Belastungen durch die Therapie, körperliche Einschränkungen und psychosoziale Folgen der Erkrankung sind Bildungschancen, freie Berufswahl sowie sportliche und soziale Aktivitäten bei Patienten mit Hämophilie Einschränkungen unterworfen. 12 13

Die Gentherapie hat das Potenzial, in den genannten Bereichen wesentliche Verbesserungen der PRO zu erzielen. Konstant höhere Spiegel des Gerinnungsfaktors könnten das Risiko schwerer Blutungen und die Entstehung oder das Fortschreiten von Gelenkschäden vermeiden. Auch die Teilhabe am sozialen Leben könnte durch den langfristigen Charakter der Gentherapie Verbesserungen erfahren.

## Methodik

Die vorliegenden Empfehlungen wurden von der GTH-Arbeitsgruppe Gentherapie erstellt und basieren auf einer systematischen Sichtung der aktuellen wissenschaftlichen Literatur mit Schwerpunkt auf den klinischen Studien zur Gentherapie der Hämophilie A und B. Persönliche Erfahrungen und Kenntnisse der Autoren aus Studien zur Gentherapie wurden einbezogen.

## AAV-basierte Gentherapie

Der heutzutage am weitesten fortgeschrittene Ansatz zur Gentherapie der Hämophilie bedient sich rekombinant hergestellter Vektoren auf der Basis des Adeno-assoziierten Virus (AAV, Abbildung 1 ). Die nicht replikationsfähigen Vektoren enthalten die cDNA für FVIII bzw. FIX unter der Kontrolle eines leberspezifischen Promoters. Die AAV-Strukturgene sowie das Signal für die Integration in das Genom wurden entfernt. Die cDNA für den Gerinnungsfaktor verbleibt überwiegend außerhalb der Chromosomen, d.h. episomal. Es handelt sich bei dem Gentransfer somit um eine additive, nicht um eine korrektive Gentherapie. 14

**Abb. 1:** Schematische Darstellung der Wirkweise einer AAV-Gentherapie. 1. Die Gentherapie wird intravenös verabreicht und mittels Endocytose in die Zelle aufgenommen. 2. Nach Endocytose enstehen aus der Zellmembran Vesikel, die Endosomen. 3. Nach Zerfall des Endosoms tritt das Kapsid über die Kernporen in den Zellkern ein. 4. Dort erfolgt die Entpackung und Freisetzung der ssDNA in extrachromosomale Episome. 5. Mittels Transkription wird die episomale DNA in mRNA umgeschrieben. Diese wird über die Kernporen in das Zellplasma ausgeschleust. 6. Im ER und Golgi-Apparat erfolgt die Translation der mRNA in Proteine. 7. Das Transgen wird in die Blutbahn sezerniert. Abkürzungen: AAV = Adeno-assoziiertes Virus; ssDNA = single-stranded DNA; mRNA = messenger RNA; ER = endplasmatisches Retikulum.

Aufgrund seines überwiegend episomalen Verbleibs wurde anfangs vermutet, dass das Transgen bei Teilung des Hepatozyten verlorengeht. Bei lebergesunden Erwachsenen scheint dies keine größere Rolle zu spielen, da in älteren klinischen Studien zur Hämophilie B über bis zu 8 Jahre stabile Faktorenspiegel beobachtet wurden. 15 Auch in präklinischen Modellen zeigte sich eine jahrelang stabile Expression. Hier wurde beobachtet, dass zufällige Vektorintegration in das Genom durchaus vorkommt, bislang jedoch nicht zu onkogenetisch relevanter Insertionsmutagenese und maligner Entartung geführt hat. 16 Langzeitdaten aus größeren Patientenkollektiven zur Beantwortung der Frage einer potenziellen Insertionsmutagenese liegen noch nicht vor.

Modifikationen des Transgens haben wesentlich zur Effektivitätssteigerung der Gentherapie beigetragen. Aufgrund der begrenzten Verpackungskapazität wird bislang eine B-Domänen-deletierte Variante von FVIII verwendet. In Vektoren für FIX wird aktuell die Variante R338L „Padua“ verwendet, die eine etwa 5- bis 10-fach erhöhte spezifische FIX-Aktivität aufweist. 17 Ein Unterschied zwischen der Gentherapie der HA und HB besteht auch darin, dass Hepatozyten nur für FIX der natürliche Ort der Biosynthese sind, während die physiologische Expression von FVIII eigentlich nicht in Hepatozyten, sondern in sinusoidalen Leber-Endothelzellen stattfindet. Dies könnte auch eine mögliche Erklärung für die unterschiedlichen Ergebnisse zur Langzeit-Expression bei Gentherapie der HA und HB sein.

Der AAV-Serotyp (variable Eigenschaften der Oberflächenstruktur) ist entscheidend für die Effizienz der hepatozellulären Transduktion und die Wahrscheinlichkeit für das Vorhandensein präformierter neutralisierender Antikörper (NA) gegen AAV beim Empfänger, die die Wirksamkeit der Therapie reduzieren können. 18 In Deutschland sind NA gegen AAV unterschiedlicher Titerhöhe bei 28% (AAV5), 43% (AAV8) und bis zu 48% (AAV2/6) der Patienten zu erwarten. 19

Zur Sicherstellung eines möglichst langanhaltenden Therapieerfolgs und zur Erfassung möglicher Nebenwirkungen ist die Nachbeobachtung nach Gentherapie von entscheidender Bedeutung. 20 Einige Wochen bis Monate nach Gentransfer kann es zu einem Anstieg der Leberenzyme, insbesondere der Alanin-Aminotransferase (ALT) kommen. Es scheint sich dabei um eine zytotoxische Immunreaktion gegen transduzierte Hepatozyten zu handeln, die AAV-Kapsid-Protein auf ihrer Oberfläche präsentieren. Auch eine unspezifische Stimulation des Immunsystems durch CpG-Oligonukleotide in der cDNA kann eine Rolle spielen. 21 Des Weiteren wird bei der Gentherapie der HA diskutiert, dass die nicht-physiologische Produktion von FVIII im Hepatozyten zu einer ALT-Erhöhung führen kann. Unbehandelt können die genannten Immunreaktionen zu einem Verlust der Expression des Transgens führen. Eine Therapie mit Glukokortikoiden, die schon bei geringem ALT-Anstieg einzuleiten ist, kann die Immunreaktion zwar eindämmen, ist aber nicht immer effektiv.

Einen Überblick über AAV-Vektoren in der klinischen Entwicklungsphase 3 gibt Tabelle 1 .

**Tabelle 1:** Gentherapieprodukte auf AAV-Basis in Phase-3-Studien

Hersteller	Bezeichnung	Serotyp Transgen	Indikation	Phase-3-Studie	Referenz (ggf. frühe Studie)
Biomarin	Valoctocogene roxaparvovec, BMN270	rAAV5 BDD-FVIII-SQ	HA	NCT03370913	Pasi et al. 22 Ozelo et al. 23
Pfizer/Sangamo	Giroctocogene fitelparvovec, SB − 525	rAAV2/6 BDD-FVIII	HA	NCT04370054	Leavitt et al. 24
CSL Behring/UniQure	Etranacogene dezaparvovec, AMT − 061	rAAV5 FIX-Padua	HB	NCT03569891	Miesbach et al. 25
Pfizer/Spark Therapeutics	Fidanacogene elaparvovec, SPK9001	rAAV8 (mod.) FIX-Padua	HB	NCT03861273	George et al. 26

Abkürzungen: AAV, Adeno-assoziiertes Virus; BDD, B-Domänen-deletiert; FVIII, Faktor VIII; FIX, Faktor IX; HA, Hämophilie A; HB, Hämophilie B; r, rekombinant.

## Zu erwartende klinische Erfolge und Risiken der Gentherapie

Eine evidenzbasierte und verständliche Darlegung von Chancen und Risiken ist für interessierte Patienten und deren Familien von größter Wichtigkeit, da Gentherapie eine grundsätzlich neue Therapieform darstellt. Eine AAV-Gentherapie kann nach heutigem Wissensstand nur einmalig angewendet werden, da nach einer Exposition die Ausbildung persistierender, hochtitriger NA zu erwarten ist. Patienten treffen mit ihrer Einwilligung zur Gentherapie deshalb eine Entscheidung, deren Langfristigkeit und Unwiderruflichkeit mit bisherigen Therapieformen nicht vergleichbar ist.

## Erfolgsaussichten

In Zulassungsstudien (einarmig, mit intraindividueller Kontrolle) werden die Veränderung von Faktoraktivität und Blutungsrate als primäre Endpunkte untersucht.

In der bereits vollständig publizierten Phase-3-Studie mit Valoctocogene roxaparvovec stieg FVIII im Mittel auf 42% an (Zeitpunkt Woche 49 bis 52). 23 Bei fast allen Teilnehmern konnte die Prophylaxe mit Faktorkonzentraten dauerhaft beendet werden. Gegenüber einem prospektiv betrachteten Kontrollzeitraum vor der Therapie ging der Verbrauch an Faktorkonzentraten um 99% und die Anzahl der Blutungen pro Jahr ( *annualized bleeding rate* , ABR) um 84% zurück. Bei den 17 Teilnehmern, die mindestens 2 Jahre nach Gentherapie untersucht wurden, lagen die mittleren und medianen Faktor VIII-Aktivitätswerte in den Wochen 49 bis 52 nach der Infusion bei 42,2 ± 50,9% bzw. 23,9% und bei 24,4 ± 29,2% bzw. 14,7% in Woche 104, was auf den sukzessiven Abfall der Faktor VIII-Aktivität hinweist. Es traten keine inhibitorischen Antikörper gegen den Gerinnungsfaktor VIII auf.

Die finalen 18-Monats-Daten der Phase-3-Studie mit Etranacogene dezaparvovec zeigen für die Gentherapie der Hämophilie B einen FIX-Anstieg auf 39% (Zeitpunkt 6 Monate) und 37% (18 Monate). 27 Der Gebrauch an Faktorkonzentraten ging in dieser Studie um 97%, die ABR um 64% zurück. In dieser Studie waren Patienten mit vorbestehenden NA gegen AAV nicht ausgeschlossen, dennoch erreichten 52 von 54 behandelten Patienten eine stabile FIX-Expression.

## Unsicherheiten und Risiken

Diesen Erfolgen stehen Unsicherheiten und Risiken gegenüber. In der Valoctocogene roxaparvovec Phase-3-Studie war die Varianz der FVIII-Expression hoch, so dass für den individuellen Patienten keine Vorhersage über erzielbare Faktorspiegel gemacht werden kann. Einige wenige Patienten wiesen vorübergehend sogar übernormale FVIII-Spiegel im Plasma auf. Auch kam es zu einem Abfall der Expression über die Zeit, weshalb zur Dauerhaftigkeit der Therapie nur orientierende Angaben gemacht werden können. Aktuell liegen Daten über den Verlauf von 6 Jahren der Phase-1/2-Studie (28) und 2 Jahren der Phase-3-Studie vor. 23

Zu einem Anstieg der Leberwerte mit Notwendigkeit einer immunsuppressiven Therapie kam es bei 85% der Patienten unter Therapie mit Valoctocogene roxaparvovec vor. 23 Die mediane Behandlungsdauer mit Immunsuppressiva betrug 230 (22-551) Tage. 71,8% der mit Glukokortikoiden behandelten Patienten entwickelten kortikosteroidtypische Nebenwirkungen.

Bei den mit Etranacogene dezaparvovec behandelten Patienten hatten 17% einen Anstieg der Leberwerte. 27 Die durchschnittliche Behandlungsdauer mit Kortikosteroiden betrug 79 Tage und war bei allen Patienten bis Woche 26 beendet.

Ansprechen und Dauer der immunsuppressiven Therapie waren somit individuell und je nach Studie unterschiedlich, weshalb auch eine Aufklärung über Nebenwirkungen einer ggf. erforderlichen längerfristigen Kortikosteroid-Medikation erfolgen muss.

Die Expression des Transgens wird nach Gentherapie nicht auf natürliche Weise reguliert. Einige Patienten haben vorübergehend eine deutlich erhöhte FVIII- oder FIX-Aktivität. Thromboembolische Ereignisse wurden in den vorgenannten Phase-3-Studien zwar nicht berichtet; ein erhöhtes Risiko kann aber nicht ausgeschlossen werden. Je ein thromboembolisches Ereignis wurde bislang im Zusammenhang mit erhöhter FVIII-Aktivität in der noch laufenden Phase-3-Studie mit Giroctocogene fitelparvovec 29 und mit erhöhter FIX-Aktivität in einer Phase-1/2-Studie mit dem AAVS3-Vektor FLT180a berichtet. 30

## Auswahl von Patienten

Die Zulassungskriterien basieren auf den Ein- und Ausschlusskriterien der klinischen Phase-3-Studien und sind der Ausgangspunkt für die Auswahl potenziell geeigneter Patienten. Sie sind produktspezifisch und unterscheiden sich zum Beispiel bezüglich des erlaubten NA-AAV-Titers. Die Testung auf NA gegen AAV ist ebenfalls produktspezifisch und erfolgt gemäß den Angaben des Herstellers, wahrscheinlich durch Einsendung in ein spezialisiertes Labor. Lebergesundheit, Infektionsstatus und Inhibitor-Vorgeschichte sind ebenfalls kritische Aspekte, die umfassend und vorausschauend beurteilt werden müssen, um optimale Patientensicherheit zu gewährleisten. Eine erfolgreich behandelte Hepatitis C-Infektion ist keine Kontraindikation für eine Gentherapie, wie auch in vielen Fällen eine gut kontrollierte HIV-Infektion. Bei einer durchgemachten Hepatitis B-Infektion ist das Risiko einer Reaktivierung unter möglicher Immunsuppression zu bedenken.

Dies gilt besonders auch für potenziell hepatotoxische Medikamente, die nach Gentherapie zwar zu meiden sind, bei Patienten mit bestimmten Vorerkrankungen aber in der Zukunft eventuell nötig werden könnten.

Neben den Zulassungskriterien spielen weitere Faktoren eine Rolle für den Erfolg der Gentherapie ( Tabelle 2 ). Um für eine Gentherapie geeignet zu sein, müssen Patienten bereit sein, sich zumindest vorübergehend auf Einschränkungen einzulassen. Sie verpflichten sich zu einem engmaschigen, anfangs mindestens wöchentlichen Monitoring von Laborwerten, ferner zur eventuell erforderlichen Einnahme von Immunsuppressiva und zu umfangreicher Dokumentation. Da Vektorbestandteile auch im Sperma ausgeschieden werden können, ist eine sichere Empfängnisverhütung im ersten Jahr nach Gentherapie obligat. Von Alkoholkonsum wird abgeraten. Damit werden Gentherapie-Patienten vorübergehend höhere Belastungen zugemutet als Patienten unter üblicher Prophylaxe mit Faktorkonzentraten oder Emicizumab. Es ist ein hohes Maß an Motivation und Zuverlässigkeit erforderlich, denn eine einmal durchgeführte Gentherapie kann nicht abgebrochen werden.

**Tabelle 2:** Aspekte der Patientenauswahl

Produktspezifische Eignungskriterien	• Alter • Schweregrad der Hämophilie • Lebergesundheit • Immunstatus bei HIV-Infektion • Inhibitor-Status • Neutralisierende AAV-Antikörper
Weitere Aspekte	• Motivation des Patienten • Beurteilung der eigenen Krankheitssituation • Individuelle Werte und Präferenzen • Klinische Blutungsneigung • Derzeitige oder künftig zu erwartende Schwierigkeiten mit der regulären Prophylaxe • Derzeitige oder künftig zu erwartende potentiell hepatotoxische Medikation • Compliance zu Therapie und Dokumentation • Familienplanung • Absehbare Belastungen durch Arztbesuche und Labor-Monitoring • Mögliche Nebenwirkungen durch Immunsuppressiva • Kosteneffektivität

Die erforderliche Compliance kann am ehesten sichergestellt werden, indem der Patient über Wirkungsweise, zu erwartende Erfolge, Risiken und Belastungen bestmöglich aufgeklärt ist. 31 Im Mittelpunkt der Betrachtung steht neben offiziellen Zulassungskriterien auch die persönliche Motivation zur Gentherapie, welche die Belastungen der ersten Monate überwinden hilft. Dabei sollten die Einschränkungen der bisherigen Therapie, Erwartungen an die Gentherapie und der Stellenwert möglicher (auch zukünftiger) Therapiealternativen systematisch und vertrauensvoll besprochen werden. Anzustreben ist eine gemeinsame Entscheidung des Patienten mit seinen behandelnden Ärztinnen und Ärzten, wobei sich die Gesprächsführung an entsprechenden Vorschlägen zum *shared decision making* orientieren sollte. 32 Schließlich ist auch zu besprechen, in welcher Form die durch die schwere Hämophilie bedingten, meist dauerhaft erreichten Nachteilausgleiche in Form eines bestimmten Grades der Behinderung (GdB) auch nach der Gentherapie weiterbestehen können.

## Anforderungen an Hämophiliezentren

Bislang ist eine Gentherapie der Hämophilie A in Europa und eine Gentherapie der Hämophilie B in den USA zugelassen. Darüber hinaus sind in Europa zwei weitere AAV-basierte gentherapeutische Ansätze zugelassen und zwar für die Behandlung der spinalen Muskelatrophie und eine Form der angeborenen Blindheit.

Während die Gentherapie für spinale Muskelatrophie (SMA) bei Säuglingen und Kleinkindern aufgrund der Schwere der zugrundeliegenden Erkrankung und des Alters der Patientinnen und Patienten während eines mehrtägigen stationären Aufenthalts verabreicht wird, 33 wird die Gentherapie für Hämophilie in Studien ambulant durchgeführt.

Die strukturellen und personellen Anforderungen zur Durchführung einer sicheren und erfolgreichen Gentherapie sind hoch und werden nicht von allen Hämophiliezentren erbracht werden können, selbst wenn sie die Kriterien zertifizierter Hämophiliezentren bereits erfüllen. 33

Der Gemeinsame Bundesausschuss (G-BA) beschloss am 14.06.2022 die Einleitung der Beratung eines Qualitätssicherungsverfahrens zur Gentherapie der Hämophilie im Rahmen der Anwendung von „Arzneimittel für neuartige Therapien“ ( *advanced therapy medicinal products* , ATMP). Die ATMP-Richtlinie wird Qualitätsanforderungen an die Gentherapie in Hämophiliezentren definieren. Da es sich um eine ambulante Therapie handelt, muss die ATMP-Richtlinie primär auf den ambulanten Sektor ausgerichtet sein. Eine umfassende Versorgungsstruktur, die bei Bedarf das gesamte Spektrum akutmedizinischer Fachdisziplinen einbinden kann, wird jedoch nur an großen sektorübergreifenden Standorten möglich sein.

Aus den Anforderungen wird sich ergeben, dass anfangs nur wenige Zentren in Deutschland, Österreich und der Schweiz die Gentherapie selbst durchführen können. Diese könnten als „Dosierungszentrum“ auch Patienten zuweisender Zentren behandeln. Die langfristige Nachsorge wird aber schon aufgrund der Anfahrtswege nicht immer am Dosierungszentrum erfolgen können, weshalb das zuweisende Zentrum auch die Funktion eines „Nachsorgezentrums“ übernehmen wird.

Diese Form der Kooperation wurde von der *European Association for Haemophilia and Allied Disorders* (EAHAD) und dem *European Haemophilia Consortium* (EHC) als „Hub-and-Spoke“ Modell beschrieben, in der das Dosierungszentrum als „Hub“ und das zuweisende und nachsorgende Zentrum als „Spoke“ bezeichnet wird ( Tabelle 3 ). 34 Hierbei handelt es sich um ein flexibles und im Prinzip veränderbares Modell, das sich an die wachsende Expertise der Hämophiliezentren anpasst. Dieses Modell ist in der Hämophilieversorgung der GTH-Länder neu und sollte zwischen den beteiligten Zentren verbindlich vereinbart werden. Das Hub-Zentrum sollte ein Hämophiliezentrum sein, das alle Aspekte der Hämophiliebehandlung anbieten kann. 35

**Tabelle 3:** Vorschlag für die Aufgabenverteilung zwischen Spoke- und Hub-Zentrum

Zentrum	Vorbereitung	Durchführung der Therapie	Nachsorge
Spoke	• Information und Aufklärung • Indikationsstellung • Eignungsprüfung (einschl. neutralisierende AAV-Antikörper) • Antrag Kostensicherung • Einwilligung Gentherapie • Einwilligung Datensammlung • Kontaktaufnahme mit Hub • Übergabe an Hub		• Monitoring von Leberwerten und Faktoraktivität • Management von späten Nebenwirkungen • Datenübermittlung an DHR, Studien und anwendungsbegleitende Datenerfassung
Hub	• Beratung • Überprüfung der Eignungskriterien	• Rezeptierung • Kontakt Apotheke • Infrastruktur für Arbeit mit gentechnisch veränderten Organismen (GVO) • Ambulante Dosierung • Management von Infusionsreaktionen und frühen Nebenwirkungen • Übergabe an Spoke	• Beratung

Voraussetzungen zur Durchführung der Vektorinfusion sind in Tabelle 4 dargestellt. Für die Zubereitung und Applikation des zugelassenen Gentherapeutikums ist keine formale Genehmigung nach Gentechnikgesetz erforderlich, es muss also kein S1- oder S2-Labor etabliert sein. Dennoch ist darauf hinzuweisen, dass neben den regulatorischen und organisatorischen Anforderungen, die für die Vektorinfusion erfüllt sein müssen, die fachliche Kompetenz in der Vorbereitung und Nachsorge mindestens ebenso wichtig für den Erfolg der Therapie ist. Die Anforderungen an das nachsorgende Zentrum (Spoke) sind deshalb ebenfalls sehr hoch und müssen entweder von diesem selbst oder durch eine geeignete Kooperationsvereinbarung mit dem Hub sichergestellt werden ( Tabelle 5 ). Es wird empfohlen, standardisierte Kooperationsverträge abzuschließen, in denen die jeweiligen Verantwortlichkeiten der kooperierenden Zentren und die Umsetzung klar definiert werden.

**Tabelle 4:** Hinweise zur Durchführung der Vektorinfusion

Dosierung erfolgt in Abhängigkeit vom tatsächlichen Körpergewicht (Vektorgenome pro kg)
Lagerung des Fertigarzneimittels vor Rekonstitution bei −60 °C
Auftauen und Rekonstitution unter Laminar-Air-Flow Arbeitsbank ohne UV-Beleuchtung
Aufbewahrung der rekonstituierten Lösung für einen begrenzten Zeitraum im Kühlschrank
Transport an den Behandlungsort mit Kühlbox
Verabreichung durch handelsübliches Infusionsbesteck mit Mikrofilter
Unterbrechung bei Unverträglichkeit während der Infusion, Gabe von Antihistaminika und Glukokortikoiden, Fortsetzung mit reduzierter Geschwindigkeit, wenn möglich
Entsorgung von Behältnissen und Infusionsbesteck über Klinikabfall

**Tabelle 5:** Anforderungen an Hämophiliezentren zur Durchführung der Gentherapie

Bereich	Anforderung	Hub	Spoke
Indikationsspezifische Kompetenz	Zertifizierung als HCCC	+	–
	Durchführung eines strukturierten Aufklärungs- und Einwilligungsprozesses	+	+ 1
Durchführung der GT	Anforderung und Bewertung des Tests auf neutralisierende AAV5-Antikörper	+	+ 1
	Kooperationsvereinbarung mit Apotheke zur Bestellung, Lagerung und Anlieferung des Fertigarzneimittels an den Behandlungsort	+	–
	Ambulante Durchführung der Infusion	+	–
	Management von infusionsassoziierten und anderen frühen Nebenwirkungen	+	–
Nachsorge und Dokumentation	Kooperation mit hepatologischem Zentrum	+	+ 1
	Tägliche Verfügbarkeit von Leberenzym- und geeigneter Faktor-Aktivitätstests mit einer Turnaround-Zeit von weniger als 24 Stunden	+	+
	Tägliche Interpretation von Laborwerten und Übermittlung von Therapieempfehlungen an den Patienten	+	+ 1
	Management von Nebenwirkungen der Immunsuppressiva	+	+ 1
	Nutzung eines elektronischen Tagebuches zur strukturierten Durchführung, Nachbeobachtung und Datenerfassung der Gentherapie	+	+
	Langfristige Datenerfassung und Übermittlung an DHR, ÖHR, SHN und andere Register	+	+

Abkürzungen: AAV, Adeno-assoziiertes Virus; DHR, Deutsches Hämophilieregister; GT, Gentherapie; HCCC, Haemophilia Comprehensive Care Centre; ÖHR, Österreichisches Hämophilieregister; SHN, Swiss Hemophilia Network.

1 Diese Aufgaben können ganz oder teilweise im Rahmen einer schriftlichen Kooperationsvereinbarung von einem Spoke- auf ein Hub-Zentrum übertragen werden.

## Interdisziplinäre Zusammenarbeit

Die Einbindung eines hepatologischen Zentrums ist erforderlich, um die Eignung von Patienten zur Gentherapie festzustellen (Beurteilung des Infektionsstatus und des Therapiebedarfs einer etwaigen Hepatitis B oder C, Beurteilung des Fibrosestadiums einer chronischen Lebererkrankung, Beurteilung potenziell erforderlicher hepatotoxischer Begleitmedikation). Ferner ist im Fall von hepatischen Nebenwirkungen einer Gentherapie die frühzeitige Einbindung der Hepatologie erforderlich. Bei Patienten mit HIV-Infektion und antiviraler Begleitmedikation ist die Einbindung der Immunologie bzw. Infektiologie erforderlich. Auch mit anderen Disziplinen kann eine enge Zusammenarbeit erforderlich sein, wenn z.B. nach Gentherapie neue Begleiterkrankungen auftreten oder potenziell hepatotoxische Medikamente indiziert sein könnten.

## Monitoring und Management von Nebenwirkungen nach Gentherapie

Nebenwirkungen unmittelbar während und nach der Infusion der Gentherapie sind selten und werden in der Regel vom Dosierungszentrum ambulant behandelt. In Ausnahmefällen kann eine stationäre Einweisung erforderlich sein, z.B. bei protrahierten oder schweren Symptomen.

Die wichtigste Nebenwirkung ist der ALT-Anstieg, der meist wenige Wochen bis Monate nach der Vektorinfusion auftritt. Da dieser asymptomatisch ist, soll die regelmäßige Kontrolle von Leberwerten und Faktorspiegeln auch ohne klinischen Verdacht erfolgen ( Tabelle 6 ). Der Therapiebedarf der asymptomatischen ALT-Erhöhung ergibt sich aus der Beobachtung, dass sie unbehandelt mit einem Verlust der Faktorexpression einhergehen kann. Bei Patienten, die mehrere Wochen nach Vektorinfusion nie eine Faktorexpression erlangt haben, ist die Therapie eines ALT-Anstiegs durch Immunsuppressiva nicht erfolgversprechend.

**Tabelle 6:** Empfehlungen zur Nachsorge nach Gentherapie

Untersuchung	Häufigkeit
• Alanin-Aminotransferase (ALT) • Faktor VIII bzw. IX (mit geeignetem Test, auch chromogen)	• <6 Monate nach GT: mindestens wöchentlich • 6 bis 24 Monate nach GT: mindestens monatlich • >2 Jahre nach GT: mindestens halbjährlich
• Aspartat-Aminotransferase (AST) • Glutamat-Dehydrogenase (GLDH) • Gamma-Glutamyltransferase (GGT) • Alkalische Phosphatase (AP) • Kreatinin-Kinase (CK) • Cholinesterase • Bilirubin	• Bei Bedarf zur Beurteilung von Differenzialdiagnosen und Schweregrad bei ALT-Erhöhung
• Gelenkstatus	• Halbjährlich
• Ultraschall und Fibroscan der Leber	• Mindestens jährlich
• Systematische Befragung zur Lebensqualität	• Jährlich

Abkürzungen: GT, Gentherapie.

Die Empfehlungen zu Start, Dosierung und Erfolgsbeurteilung der immunsuppressiven Therapie bei ALT-Anstieg sind derzeit noch im Fluss und werden in weiteren klinischen Studien untersucht. Die GTH-Arbeitsgruppe Gentherapie wird die Datenlage beobachten und ihre Empfehlungen danach aktualisieren. Auch wird auf die Fachinformationen der Hersteller verwiesen.

Als Anhaltspunkte schlagen wir vor, eine immunsuppressive Therapie zu starten bei:

- Erhöhung der ALT oberhalb des Normbereichs oder
- Anstieg der ALT auf mehr als das 1,5-fache des Ausgangswerts oder
- Abfall der Faktoraktivität um mehr als ein Fünftel (20%) der Vorwerte

Ausgangswerte der ALT sollten möglichst als Mittelwert von 3 Werten vor Gentherapie bestimmt werden, die Vorwerte der Faktoraktivität als gleitender Durchschnitt der letzten 3-5 Werte nach Gentherapie. Da die Bestimmung der Laborwerte starken methodischen Schwankungen unterliegt, sollte sie möglichst immer im selben Labor unter Verwendung der gleichen Geräte und Reagenzien erfolgen. Bei Umstellungen der Methodik sind Ausgangs- bzw. Vorwerte neu zu beurteilen. Da Transaminasen einer zirkadianen Rhythmik unterliegen, sollte die Bestimmung möglichst zur gleichen Tageszeit erfolgen. Abweichungen davon sowie auch Änderungen der Medikation, Ernährungsgewohnheit und Alkoholzufuhr müssen in die Beurteilung einbezogen werden. Ebenso ist ein später Anstieg der ALT, z.B. mehr als 1 Jahr nach Gentherapie, nicht unbedingt als Zeichen einer zellulären Immunreaktion gegen transduzierte Zellen zu werten, sondern könnte andere Ursachen haben.

Wenn eine immunsuppressive Therapie erforderlich wird, schlägt die GTH im Einklang mit den Phase-3-Studienprotokollen vor, mit Prednisolon 60 mg pro Tag oral (diese Dosis kann nach Körpergewicht angepasst werden) oder der äquivalenten Dosis eines anderen Glukokortikoids zu starten. Die Therapie sollte zunächst für einen Zeitraum von mindestens 14 Tagen gegeben und dann je nach Verlauf fortgeführt oder schrittweise unter weiterer Beobachtung der Laborwerte ausgeschlichen werden:

- Beginn der Dosisreduktion, wenn die ALT um 50% oder auf den Ausgangswert abgefallen ist. Eine vollständige Normalisierung muss in der Regel nicht abgewartet werden.
- Reduktion auf Prednisolon 40 mg, 20 mg, 10 mg oder auf die äquivalente Dosis eines anderen Glukokortikoids für jeweils eine Woche, danach
- Reduktion auf Prednisolon 7,5 mg, 5 mg und 2,5 mg oder auf die äquivalente Dosis eines anderen Glukokortikoids für jeweils eine Woche, dann absetzen.

Sollte es während des Ausschleichens zu einem erneuten ALT-Anstieg um mehr als das 1,5-fache kommen, wird empfohlen, auf die zuletzt wirksame Dosis zu erhöhen und nach 14 Tagen einen neuen Ausschleichversuch zu starten. Ist eine Prednisolon-Therapie mit mehr als 10 mg pro Tag oder Äquivalent für mehr als 1 Monat erforderlich, sollte eine Osteoporose-Prophylaxe mit Kalzium und Vitamin D _3_ in Erwägung gezogen werden. Bei Risikofaktoren für Gastritis oder gastrointestinale Blutungen sollte ein Protonenpumpen-Inhibitor gegeben werden. Die Prophylaxe einer Hepatitis B-Virus (HBV)-Reaktivierung bei Patienten mit durchgemachter HBV-Infektion ist zu bedenken.

Gelingt das Ausschleichen einer Prednisolon-Therapie auch im zweiten Versuch nicht, sollte über eine Umstellung von Prednisolon auf Budesonid oder den Einsatz einer kortikosteroidsparenden Zweitlinientherapie entschieden werden. Dafür kommen Tacrolimus, Azathioprin oder Mycophenolat-Mofetil in Betracht, die zunächst in Kombination mit dem Kortikosteroid und danach allein gegeben werden. Aktuelle Studien untersuchen den prophylaktischen Einsatz von Immunsuppressiva, um eine ALT-Erhöhung von vornherein zu vermeiden. 30

Eine Untersuchung auf Ausscheidung von Vektor-DNA ist in klinischen Studien erfolgt. Hier zeigt sich, dass in Körperflüssigkeiten (Urin, Stuhl, Speichel, Samenflüssigkeit) noch mehrere Monate nach Durchführung der Gentherapie Vektorbestandteile nachgewiesen werden können. 36 In der Routine sind Untersuchungen auf dieses *vector shedding* nicht erforderlich. Es besteht kein Bedarf für Isolationsmaßnahmen im häuslichen Bereich, jedoch ist eine physikalische Kontrazeption (Kondome) empfohlen.

Tumorerkrankungen sind bislang bei vier Patienten nach Gentherapie aufgetreten. 21 37 38 39 Die umfassende klinische, histopathologische und molekulargenetische Aufarbeitung dieser Fälle ergab keinen Anhalt für einen kausalen Zusammenhang mit der Gentherapie. Auch in künftigen Fällen von Tumoren nach Gentherapie ist eine umfassende molekulargenetische Aufarbeitung zu veranlassen.

## Nutzung elektronischer Anwendungen

Die GTH empfiehlt die Nutzung geeigneter elektronischer Systeme, um den besonderen Herausforderungen der Gentherapie gerecht zu werden. Dies betrifft zunächst die Eignungsphase, in der essenzielle körperliche, apparative und Laboruntersuchungen dokumentiert werden müssen. Checklisten können helfen, vor einer Vorstellung des Patienten im Dosierungszentrum (Hub) die erforderlichen Befunde und sonstigen Unterlagen im zuweisenden Zentrum (Spoke) zu sammeln. 40 Diese werden in der Anwendung wie in einer Akte dokumentiert und mit Einverständnis des Patienten auch schon vor einer Vorstellung im Hub-Zentrum mit diesem geteilt. So können unnötige Doppeluntersuchungen und Fahrten in das Dosierzentrum reduziert werden.

Spätestens mit der Dosierung der Gentherapie soll eine dafür geeignete elektronische Dokumentationshilfe verwendet werden, in der alle relevanten Informationen von Hub-Zentrum, Spoke-Zentrum und Patient gemeinsam dokumentiert werden. Elektronische Tagebücher sind schon heute in der Dokumentation der Hämophilie-Behandlung üblich und werden von vielen Patienten gewissenhaft geführt. 41 42 43 Es ist deshalb sinnvoll, den Patienten in die Dokumentation aller relevanten Daten aktiv einzubinden. So können vor allem in der Nachsorge auch Informationen zu Blutungen und anderen PRO ohne Zeitverlust allen beteiligten Ärztinnen und Ärzten vorliegen und helfen, den Patienten optimal zu beraten. Die Einbindung des Patienten in das Dokumentationssystem kann auch den Informationsfluss an den Patienten sichern helfen, wenn z.B. die aktuell empfohlene Begleitmedikation in Echtzeit zur Verfügung gestellt wird und sichtbar ist, welches Zentrum (Hub oder Spoke) aktuell die Therapiesteuerung innehat, wer für Fragen zuständig und im Notfall erreichbar ist. Dies erhöht die Patientensicherheit und fördert die langfristige Compliance nach Gentherapie. Elektronisches Termin-Management und andere Werkzeuge können diesen Aspekt zusätzlich unterstützen.

Besonders nützlich ist eine von Hub, Spoke und Patient gemeinsam genutzte elektronische Plattform für die Steuerung der immunsuppressiven Therapie. Idealerweise sollten beide Zentren die dafür relevanten Laborwerte in Echtzeit einsehen und zur Entscheidungsfindung beurteilen können. Nützlich könnte die Visualisierung gleitender Durchschnitte und prozentualer Abweichungen vom Ausgangswert sein, um einen nachvollziehbaren Entscheidungsprozess zu unterstützen.

Nicht zuletzt werden elektronische Informationssysteme zum Management der Gentherapie auch die wesentliche Primärquelle für Daten sein, die für Register und Studien benötigt werden. Diese umfassen unter anderem:

- Postmarketing-Studien pharmazeutischer Unternehmer gemäß Zulassungsauflagen der europäischen Arzneimittelbehörde
- Anwendungsbegleitende Datenerhebung, wie sie vom G-BA festgelegt wird
- Deutsches Hämophilieregister (DHR), Österreichisches Hämophilieregister (ÖHR), SHN-Register (Swiss Hemophilia Network)
- Beobachtungsstudien und internationale Register wie das Register der Welt-Hämophilie-Organisation (WFH). 43

Neben der inhaltlichen Planung und Programmierung einer solchen elektronischen Plattform sind Datenschutz, Datensicherheit, Finanzierung und vertragliche Regelung zwischen den beteiligten Einrichtungen nicht unerhebliche Herausforderungen. 45 Es ist eine lebenslange Dokumentation nach Gentherapie zu empfehlen.

Am weitesten fortgeschritten ist das globale Gentherapie-Register des WFH (GTR-WFH).

## Ausblick Finanzierung der Gentherapie

Die Gentherapie verursacht zum Zeitpunkt der Dosierung einmalig sehr hohe Kosten, die durch Einsparungen in den folgenden Jahren ausgeglichen werden. Klinische Studien zeigen, dass der Faktorverbrauch bereits im ersten Jahr nach Gentherapie um bis zu 98% sinkt.

Die Gentherapie ist als ambulante Therapie zugelassen und als solche erstattungsfähig. Im Bereich der gesetzlichen Krankenversicherung (GKV) in Deutschland haben Krankenkassen die Möglichkeit, mit pharmazeutischen Unternehmen (pU) Rabattierungsverträge nach § 130a SGB V abzuschließen und dabei ein „Pay-for-Performance“ (P4P)-Vergütungsmodell nach § 130a Abs. 8 SGB V (messbare Therapierfolge) zu implementieren.

Auf Seiten der ärztlichen Leistungserbringer sind die Vorbereitung, Durchführung und Nachsorge der Gentherapie mit erheblich höherem Aufwand als die übliche Hämophilieversorgung verbunden. Die für den Therapieerfolg entscheidende Qualität der Behandlung sollte über Versorgungsverträge vereinbart und vergütet werden, wofür der § 132i SGB V einen Rahmen bieten kann. Alternativ kommen Versorgungsverträge nach § 140a SGB V „Besondere Versorgung“ in Betracht. Durch adäquate Finanzierungsmodelle kann die fehlende Abbildung der ärztlichen Versorgungsleistung im einheitlichen Bewertungsmaßstab (EBM) bzw. in der Gebührenordnung für Ärzte (GOÄ) ausgeglichen werden.

## Ausblick Pädiatrie

Obwohl Gentherapiestudien im Kindesalter bereits seit den 1990er Jahren bei seltenen monogenen und potenziell tödlichen Erkrankungen durchgeführt wurden, haben Komplikationen mit maligner Entartung und Todesfällen dazu geführt, dass diese Therapieform Ängste und Sorgen bei betroffenen Familien hervorruft und zum Teil negativ behaftet ist. In den letzten Jahren wurden mit AAV-basierter Gentherapie der spinalen Muskelatrophie und mit lentiviraler Gentherapie hämatopoetischer Stammzellen zur Behandlung der transfusionsabhängigen Thalassämie Erfolge erzielt.

In der Hämophilie sind Studien zur Behandlung von Kindern und Jugendlichen mit AAV-Vektoren in Planung. Dabei müssen einige Limitationen wie die hohe Prävalenz von NA gegen AAV und das im frühen Kindesalter noch nicht abgeschlossene Wachstum der Leber berücksichtigt werden. Eine Wiederholung einer AAV-Gentherapie im späteren Lebensalter ist nach derzeitigem Wissensstand nicht möglich.

Im Rahmen des *shared decision making* benötigen nicht nur Patienten, sondern auch ihre Sorgeberechtigten fundierte und verständliche Informationen und Beratung im Hinblick auf die Gentherapie. Die Aufklärung der Sorgeberechtigten minderjähriger Patienten ist dabei besonders anspruchsvoll, weil Stellvertreter-Entscheidungen für einen Minderjährigen getroffen werden müssen. Gleichwohl ist die Aussicht auf eine möglichst frühzeitige Gentherapie der Hämophilie auch mit der Hoffnung verbunden, die eingangs geschilderten körperlichen und psychosozialen Nachteile der Erkrankung besonders nachhaltig zu korrigieren.

## Zusammenfassung und Ausblick

Die Gentherapie hat das Potenzial, patientenrelevante Endpunkte der bisherigen Therapien zu übertreffen. Es bestehen jedoch weiterhin eine hohe Variabilität des Ansprechens und eine teilweise hohe Nebenwirkungsrate mit ALT-Erhöhung und der Notwendigkeit einer immunsuppressiven Therapie. Daher sind Auswahl und Beratung geeigneter Patienten von großer Bedeutung. Ein gründliches und effektives Management von Nebenwirkungen, insbesondere der Anstieg von Leberwerten, ist entscheidend für den Erfolg der Therapie. An die Hämophiliezentren werden neue Anforderungen durch Qualitätsrichtlinien formuliert, die durch Kooperation der Zentren (Hub-and-Spoke Modell) und interdisziplinäre Zusammenarbeit erfüllt werden können. Moderne elektronische Systeme werden die Kooperation der beteiligten Zentren und die Einbindung des Patienten unterstützen. Gleichzeitig helfen sie, wichtige Daten longitudinal zu sammeln, die bei der Beantwortung offener Fragen und zur Nutzenbewertung der Gentherapie helfen. Die Gentherapie stellt auch neuartige Anforderungen an die Finanzierung durch die GKV. Für Hämophilie-Patienten im Kindesalter ist die Gentherapie heute noch keine Option, auch wenn klinische Studien dazu in Planung sind.

**Erratum** Please note: this article has been changed into an open access article.
